# USING SPATIAL ANALYSIS TO OPTIMIZE DISASTER PREPAREDNESS FOR FRAIL OLDER ADULTS

**DOI:** 10.1093/geroni/igz038.3239

**Published:** 2019-11-08

**Authors:** Megan Bond, Jessica L Tice

**Affiliations:** Florida Department of Elder Affairs, Tallahassee, Florida, United States

## Abstract

The Florida Department of Elder Affairs (DOEA) provides programs and services for over 65,300 older people and adults with disabilities. These individuals are uniquely vulnerable and may be disrupted, displaced, and disoriented during the natural disasters common to Florida. DOEA clients are homebound or dependent upon community-based services to provide supervision or direct assistance to perform basic self-care. Often, clients are unable to complete personal care independently, which makes sheltering in place a unique challenge, yet they can only evacuate with special transportation and support arrangements in the shelter. It is critical to DOEA to accurately predict clients likely to be seriously affected by storms, plan for relocation before an event, and arrange for the provision of extended care after. DOEA responded by utilizing ArcGIS mapping software to join client residence locations to evacuation zone polygons and developed a methodology to prioritize clients with personal and functional barriers to evacuation. Proven during Hurricane Michael (2018), local emergency managers were able to use this tool to complete wellness checks on survivors before outside aid arrived. This initiative is evolving with the challenges posed by each storm season. Hurricane Dorian (2019) required the addition of latitude and longitude of client locations for when traditional street navigation became unavailable. Importing and overlaying primary data on secondary emergency management resources is a strategy that could be replicated by other organizations that have similar needs to reconcile individual locations in context of local threats, making this methodology transferrable to other disaster and flood-prone communities.

